# PReS-FINAL-2274: Antiadalimumab antibodies in pediatric rheumatology patients. A pilot experience

**DOI:** 10.1186/1546-0096-11-S2-P264

**Published:** 2013-12-05

**Authors:** S Murias Loza, RM Alcobendas Rueda, A Remesal Camba, D Pascual Salcedo, J Peralta, R Merino Muñoz

**Affiliations:** 1 Pediatric Rheumatology, Hospital Universitario La Paz, Madrid, Spain; 2 Immunology, Hospital Universitario La Paz, Madrid, Spain; 3 Ophthalmology, Hospital Universitario La Paz, Madrid, Spain

## Introduction

Immunogenicity of anti-Tumor Necrosis Factor agents is one of the mechanisms behind treatment failure.

## Objectives

To explore the relationship between the presence of antiadalimumab antibodies (AAA), the disease activity and the therapeutic decisions.

## Methods

Cross-sectional retrospective study determining: 1) serum adalimumab (Ada) levels by a capture ELISA (positive > 5 ng/ml) and 2) levels of AAA by a two-site bridging ELISA [positive > 10 Arbitrary Units (AU)/ml]. The clinical activity was assessed by conventional tests and the physician visual analogue scale (ph-VAS) of 0 to 10, the same day that the blood sample was collected. Blood samples were obtained a few hours before drug administration.

## Results

Until March 2013 measurements in 25 patients were available, 15 (60%) of them had some activity of their disease ph-VAS = 1.7 ± 0.9 (1-4). Eight (32%) children presented AAA, range from 12 to 30,000 AU/ml, and absence of Ada levels. All cases with AAA positives had active disease, except one who had received a periocular corticosteroid injection in the previous month. Furthermore 8/17 (47%) patients without AAA had active disease. The duration disease and the age were not different between those with and without AAA. The only clinical and analytical difference was the higher frequency of active uveitis when AAA were present (p = 0.01). Similarly median survival time on treatment was shorter when AAA were present (2.3 *vs *2.5 years) (p = 0.03).

## Conclusion

Antiadalimumab antibodies appear to explain half of the cases of active disease and their presence is associated with discontinuation of treatment.

## Disclosure of interest

None declared.

**Table 1:** Characteristics of 25 children with pediatric rheumatic diseases treated with Adalimumab.

AAA	Girls	JIA	ANA+	Uveitis	Active uveitis	Active arthritis	MTX	Drug switching
Present n = 8	6 (75)	4 (50)	4 (50)	8 (100)	6 (75)	2 (25)	2 (25)	4 (50)
Absent n = 17	13 (77)	15 (88)	6 (35)	13 (77)	3 (18)	5 (29)	6 (35)	4 (24)

Data are expressed as n (%)

AAA = Antiadalimumab antibodies; JIA = Juvenile Idiopathic Arthritis; ANA = Antinuclear antibodies; MTX = Concomitant Methotrexate.

